# Hospital Practice in Vienna

**Published:** 1886-11

**Authors:** Frank Billings

**Affiliations:** Lecturer on Physical Diagnosis in the Chicago Medical College; 235 State Street


NOTES ON HOSPITAL PRACTICE IN VIENNA. *Abstract of a paper by* FRANK BILLINGS, M.D., *Lecturer on Physical Diagnosis in the Chicago Medical College.*

[Read before the Chicago Medical Society, October 18, 1886.]

In the medical wards of Professor Nothnagel, the temperature of important cases is taken every two hours by the nurse, by placing a non self-registering thermometer of the centigrade scale in the axilla for five minutes. From the temperature markings charts are made and kept at the heads of the beds.

Examination, chemical and microscopical, of the urine is made of all cases upon admission to the hospital, and daily examination of important cases.

The tests used are the following:

For albumen: the heat test, and the acetic acid and ferrocyanide of potassium test.

For peptone: sulphate of copper and hydrate of potassium (as in Trommer's test for sugar), giving a violet color if peptone is present. Albumen must be proven to be *absent* by the above two tests before making the test for peptone, for albumen gives the same reaction to the peptone test.

For blood: the addition of potassium hydrate and heating precipitates phosphates, which are colored red or reddish when blood is present. Microscopic and spectroscopic examinations are also made in doubtful cases.

For bile: hydrochloric acid and hydrate of potassium are added to urine, in about equal amounts, giving a green color to the whole mixture when bile is present.

The well-known nitric acid test is also used.

For indican: to urine is added a small amount of hydrochloric acid, one drop of a five per centum solution of hyposulphite of lime and a small amount of chloroform; the test-tube is then shaken and the chloroform allowed to settle to the bottom of the tube. When indican is present the chloroform will have a blue or bluish tint. The presence of iodides in urine of a patient undergoing treatment with iodine or iodides will give a violet or reddish tint to the chloroform, in the above test.

For sugar: Moore's, or Trommer's tests are used. In doubtful cases the Fischer-Jaksch test is employed. It consists in dissolving phenyl hydrocene (a chlor-hydrate of phenyl) gram 1, sodium acetate gram .750 in water 10 c. c. in a test-tube by warming and adding about 25 c. c. of the suspected urine. The mixture is evaporated to one-half its bulk over a water or sand bath, and a precipitate occurs if sugar be present. Microscopically, this precipitate is seen to be composed of yellowish or yellowish-brown, acicular crystals, arranged in fan-shaped forms. It will detect gram .001 of sugar in 1000 c. c. of urine, *i. e.*, one millionth of one per centum.

The presence of aceto-acetic acid, *Diacetone*, is considered of great importance in prognosis of continued fevers, eruptive fevers, diabetes mellitus, and pyæmia or septicæmia.

To prove the presence of aceto-acetic acid it is necessary to first find acetone in the urine,—a body always present in high fevers, according to Dr. von Jaksch.

To find acetone, add to the urine a sufficient amount of sodium nitro-prusside to give a Bordeaux color; then add strong acetic acid, drop by drop, which gives darker rings, where the acid comes in contact with the fluid, if acetone be present.

Or, distill the urine; add a small amount of iodine, iodide of potassium and hydrate of potassium; heat, and a precipitate, or odor of iodoform will occur if acetone be present, Having proven acetone to be present, add to the urine the tincture of the chloride of iron. This gives a violet color if diacetone be present. This color disappears upon the application of heat, or upon standing for twenty-four hours. The presence of salicylates in the urine gives the same color upon addition of tincture of iron, but the color does not disappear upon heating the urine, or in twenty-four hours.

*Diacetonurea* indicates a grave or guarded prognosis.

Tuberculosis in some form is very common in Vienna. Tubercular lesions are found in nearly seventy per centum of the deaths! It masks and complicates other diseases and occurs so frequently that it is called *Mortuis Vienensis.* In cases where physical signs are insufficient to arrive at a diagnosis, the presence of tubercular bacilli in the sputa, urine, feces or discharge from a wound, is taken as positive evidence of tuberculosis.

Until 1878 or '79, Vienna secured water from the Danube. Since that time the water is brought by aqueduct from mountain springs about eighty miles away. Typhoid fever was endemic and at times epidemic when Danube water was used. Dr. Jaksch is authority for the statement that not one case of typhoid fever has developed within the city since the new water supply was completed.

A few cases of typhoid fever, brought from the surrounding country, are to be seen in the wards.

The treatment of typhoid fever is expectant. Cold baths of 75° to 80° F., and when this is not sufficient as an antipyretic, an occasional dose of quinia, antipyrin or thallin is used.

During the stage of *lysis* the same medical antipyretics are used more continuously to make the fever abate more rapidly.

Acute articular rheumatism is treated by the administration of salicylic acid gram. 10 to 24 in the first twenty-four hours; half that quantity daily for the next three or four days, and then gradually diminishing doses. Salicylate intoxication occasionally occurs, manifested by delirium and erythema of the skin.

Large doses of benzoate of sodium; gram. 20 to 30, even gram. 60, daily, are also used in rheumatism. Lobar pneumonia receives no antipyretic treatment whatever,—and often no other treatment. Alcohol is given freely to sustain the heart when necessary, and wet-cupping or leeching over the affected lobe for *pain.* Wet-cupping or leeching is often practiced for an anodyne effect in local inflammation,—perityphlitis pleurisy, local peritonitis. Urethan is used as a hypnotic in place of chloral, or morphine. In cases of enfeebled and irregular heart-action due to valvular disease, myocarditis, etc., nitro-glycerine in doses of gram .001 every four hours, or caffeine citrate or salicylate gram 1.0 daily, are used.

In cases of dyspepsia from weak or irregular heart-action, due to valvular disease, adhesive pericarditis, etc.; with chronic venous congestion of liver and kidneys, scanty urine, and general dropsy, as a result, small and frequently repeated doses of calomel are given to increase the amount of urine.

In the obstetrical wards of Professor Carl Braun one large room is used for delivering patients. Each student or physician practicing in the ward is allowed to touch but one patient until her labour is completed. The hands of those practicing in the ward are rendered aseptic, by a thorough washing and brushing, and then immersed in a one per centum solution of permanganate of potassium; they are rendered still more aseptic and bleached by an immersion in a ten per centum solution of hydrochloric acid.

Multipara, in normal labour, are allowed to assume any position they choose in the second stage, and but little care is taken to support the perineum.

Primipara are placed upon the left side, and the perineum is carefully supported by the well-known German method.

In normal cases vaginal douches of two per centum watery solution of carbolic acid or corrosive sublimate, 1 part to 4,000 of water, are used immediately after labour and daily thereafter until discharged.

In all cases where the hand or instruments are inserted into the uterus and in cervical or perineal lacerations, after closing the rents an intra-uterine douche of one of the above named solutions is given immediately, but is not repeated unless fever, or offensive lochia occur; a daily vaginal douche only being given. Puerperal convulsions are treated by immersion in hot bath for a variable length of time; rectal injections of chloral gram 1.0 are given, and repeated after each convulsion, and also hypodermic injection of morphine. As a rule no attempt is made to bring on labour by artificial dilatation of the os uteri. I saw one case treated as above; labour occurred in twenty-four hours, resulting favorably to the mother and child.

Abortion is treated upon the principle of non-interference. Rest in bed is enjoined until the embryo presents itself in the cervical canal, when it is enucleated by inserting a Ferguson's speculum; the cervix being received within the proximal end of the speculum, then by upward pressure upon the speculum and counter-pressure upon the fundus uteri from the hypogastrium the ovum is forced into the speculum intact. If hæmorrhage occur a colpeurynter is inserted, and ergot is sometimes given. If the embryo is discharged and the placenta remain no interference is practiced for three or four days—until partial separation by fatty degeneration has occurred—when it is partially or wholly removed by enucleation as described above, or by the finger or forceps. If sepsis and fever call for interference, the placenta is wholly or partially removed by forceps and the finger and intra-uterine douches given.

Four cases of corrosive sublimate poisoning occurred last year: two each in the wards of Professors Carl and Gustavus Braun. The solution used was 1 part to 4,000 of water. All died with symptoms of inflammation throughout the whole alimentary tract. The autopsies revealed ulceration throughout the upper portion of the tract, with blackened, necrosed mucous membrane, almost gangrenous in the colon and rectum.

In the surgical wards of Professors Billroth and Albert the smallest details of cleanliness are scrupulously practiced. The operating-room of Professor Billroth is aired and cleaned daily. The floor is so constructed that all liquids flow to the center of the room and pass away through a grating. The floor is scrubbed and douched thoroughly every day. Two reservoirs are placed high on the wall, containing a one per centum solution of carbolic acid, and a rubber tube attached to it carries the solution to any part of the arena for irrigating wounds. The operator as well as assistants and nurses are clothed in fresh linen, and having a long ulster of linen worn outside. Their hands are thoroughly brushed and washed in a one per centum solution of carbolic acid. The patient is prepared by a bath and fresh clothing. The part to be operated upon is thoroughly scrubbed, shaved and irrigated with a one per centum carbolized solution. Sponges are thoroughly cleaned and bleached, and, with all towels, napkins and silk sutures are boiled for one hour in a five per centum solution of carbolic acid, and are then placed in a clean five percentum solution of carbolic acid for *fourteen* days, the solution being changed every two to four days before use. During an operation all instruments are kept in a two and one-half per centum solution of carbolic acid. Urethral and uterine sounds, clamps and other non-cutting instruments used in laparotomy are kept in a ten per centum solution of carbolic acid in glycerine during the operation, but are dipped into a two and one-half per centum watery solution immediately before use.

Cutting instruments are thoroughly rubbed after cleaning, and, if used about infecting pus, etc., are heated in flame and sent to polisher before being used again. Rubber drainage-tubing is soaked in warm water for one day, then placed in a five per centum carbolized solution for two or three days, when they are changed to a fresh solution, and are fit for use after two weeks. The carbolized solution is changed every two weeks. A solution of corrosive sublimate one part to 1,000 to 2,000 of water is used for irrigating wounds in place of the one per centum carbolized solution.

Powdered iodoform or boracic acid is placed over a closed wound or packed into an open one. Iodoform, carbolized or sublimated gauze is used next to the wound with borated or absorbent cotton, impervious mackintosh and a plain or aseptic roller bandage over all. An adhesive iodoform gauze, impregnated with colophonium, a resinous residue in distillation of turpentine, is used in operations about the face, where parenchymatous hæmorrhage occurs.

Other antiseptic preparations used in the clinic are: iodoform plaster, a mixture of iodoform, glycerine and mucilage of gum arabic, spread upon linen, for covering abrasions and for purposes for which ordinary adhesive plaster is used; iodoform glycerine, 10 to 20 parts to 100, for injecting abscesses after aspiration; iodoform collodion, 1 part to 10, for covering abrasions and superficial wounds; iodoform bacilli, consisting of idoform 20 parts, pulvis acacia, glycerine amylum, of each 2 parts, made into bacilli of different sizes for insertion into fistulæ and deep wounds which cannot be irrigated or well cleansed.

Professor Kaposi treats lupus by repeated application of fused nitrate of silver to the tubercles, the stick of nitrate of silver being gouged deeply into the diseased tissue.

Syphilis, in the first and second stages, is treated by inunction of unguentum hydrargyri; gram 2.0 to 4.0 daily for twenty-four successive days, being rubbed into the skin of back, abdomen and inner surfaces of extremities. A solution of potassium chlorate is used to allay any tendency to stomatitis mercurialis. Mucuous patches are touched with lunar caustic, and covered with spray of iodoform in ether.

From 2,500 to 3,000 deaths occur in the hospital annually. Autopsies are held upon all. Tubercular lesions predominate. Abscesses and echinococcus cysts of the liver are often seen. Amyloid degeneration of the liver, spleen and kidneys is common, and amyloid degeneration of muscles, especially of the heart, is occasionally seen.

Pure bacterial cultivations are made from the bodies of those dead from typhoid fever, pneumonia, erysipelas, septicæmia, pyæmia, glanders and tuberculosis. Experimental inoculations are made upon lower animals from these cultivations.

Bacteriological laboratories are also maintained in the departments of surgery, medicine, skin diseases, specific diseases, and in the polyclinic.

235 State Street.

